# Hybrid meat products and co-creation: What do consumers say, feel and think?

**DOI:** 10.3389/fnut.2023.1106079

**Published:** 2023-02-16

**Authors:** Chris Ryder, Sylvia Jaworska, Simona Grasso

**Affiliations:** ^1^Department of English Language and Linguistics, University of Reading, Reading, United Kingdom; ^2^School of Agriculture and Food Science, University College Dublin, Belfield, Ireland

**Keywords:** consumer co-creation, word associations, evaluation, hybrid meat products, cross-cultural, United Kingdom, Spain, Denmark

## Abstract

**Introduction:**

What consumers say about food and what kind of words they use to do so offers direct insights into their perceptions, preferences, reasoning, and emotions.

**Methods:**

This study explores evaluations of hybrid meat products of 2,405 consumers from England, Denmark, and Spain. As part of a large survey, consumers were prompted to note down four words that come to mind when they read a description of a hybrid meat product, and then again after they were involved in a hypothetical co-creation task of a hybrid meat product. 18,697 words and phrases of language material was processed using computational corpus-based analysis and manual classification into semantic categories including: Evaluation, Sensory, Production, Emotion, Diets, Quality, Ethics, and Other.

**Results:**

Consumers consider many dimensions when it comes to the evaluation of hybrid meat products including ethical conduct and sustainability. For all three languages, the number of positive words increased and the number of negative words decreased significantly (*p* < 0.001) following the co-creation task, suggesting that consumers see such products very positively once they are more familiar with them and know more about the ingredients. Subcategories that received most words include: taste, ingredients, healthiness, naturalness, innovation, and environment, implying that these areas are of most importance when it comes to the evaluation of hybrid meat products. The concept of nutrition (especially words pointing to positive aspects such as “rich in vitamins”, “nutritious”) also rose significantly in use after co-creation.

**Discussion:**

The study reveals consumers’ vocabulary of hybrid meat products across the three countries and offers important insights for food producers to help them create innovative products that better align with consumers’ perceptions and expectations.

## 1. Introduction

Flexitarian eating has been on the rise in recent years and seems to have accelerated post the Covid-19 pandemic and in younger populations referred to as Gen Z. According to the recent YouGov data, one in five 18-to-24-year-olds currently follow such a diet and the number has doubled since 2019 (1). Flexitarian diet includes both: reducing meat consumption and meat portion size along the week, and having more plant-based and meat-free meals (2). In this context, a diversity of hybrid meat products have been introduced on the market; these products are specifically designed to blend meat and plant-based ingredients in convenient ready to cook forms, such as burgers, sausages or minced products (3). Despite the novelty of this hybrid concept, some consumer studies have already been carried out (4–6), and recent research has focussed on understanding consumer attitudes toward such products, including consumers’ views on their formulation and different types of messaging on them (7, 8).

Since consumers are an essential part in the new product development process and their attitudes and views can increase (or decrease) the likelihood of success of a new product on the market, co-creation has been suggested as a valuable tool to understand consumers’ concerns and perceptions, and engage them in the design of new food products (9). For example, consumer co-creation has been recently applied in a cross-country study involving consumers from United Kingdom, Spain, and Denmark, with the aim to understand the preferred ingredients to use in the manufacturing of hybrid meat products (8). A comparison of the consumption habits for processed meat in these three countries, to each other as well as to Europe and worldwide, can be seen in Table 1. Note that “Processed Meat” in this sense includes cold and roast meat products, ham and bacon, and sausages; fresh meat and pre-cooked meat-based ready-to-eat products are not included.

**TABLE 1:** Average volume (kg) per capita for estimated 2023 consumption of processed meat.^a^

Region	Cold and roast meat	Ham and bacon	Sausages	Total
UK	8.2	2.1	2.7	12.9
Spain	4.4	2.7	4.1	11.2
Denmark	7.1	2.6	8.6	18.3
Europe	18.8	7.2	3.2	7.4
Worldwide	2.3	0.8	1.9	5.1

^a^Source: Statista (15).

In the present study and covering the same countries, we use a linguistic word analysis approach to compare consumer attitudes toward hybrid meat products under two different conditions: the first condition exposed consumers to a written definition of hybrid meat products and the second invited them to a participative co-creation task of hybrid meat products, in which they could build their preferred product in a series of short steps. Following each condition, participants were prompted to provide any four words about the product that came to mind. Subsequently, the words were analysed systematically using frequencies and semantic categorisation.

This kind of free word association analysis is a very powerful tool that can tap into consumers’ involuntary, and therefore more authentic preferences, expectations, reasoning and emotions when it comes to evaluating novel food products [e.g., (10–12)]. This is important to understand since most of the consumers’ everyday decision making is determined by a constellation of spontaneous experiential, affective, and reasoning factors (13), and words that come to mind spontaneously can reflect these constellations unlike experiments conducted in laboratory settings. What consumers think spontaneously about hybrid meat products can therefore help producers of such products understand why certain products are preferred over others, and therefore more likely to be purchased. Research has shown that the understanding of this kind of free and spontaneous word associations facilitate an effective food product development and can assist with successful introduction of novel of healthy foods [e.g., for a systematic review on this research see (14)]. Furthermore, since co-creation has been shown to increase the likelihood of success of novel food products, word association analysis post a co-creation task can further enhance our understanding of the role of consumer engagement in novel food creation, and provide food manufacturers with relevant information to help them align the development of hybrid meat products with consumers’ preferences and expectations. This study therefore aims to explore the following research questions:

1. How do consumers perceive and evaluate hybrid meat products upon first presentation?
2. How do these perceptions change following the co-creation of a hybrid meat product?
3. In what ways does the linguistic and cultural context affect perceptions of hybrid meat products both before and after co-creation?

This study takes an innovative approach to the examination of hybrid meat products, firstly due to its foundations in Grasso et al. (8) pioneering study into the co-creation of hybrid meat products in the United Kingdom, Spain, and Denmark, and secondly by building on the corpus-linguistic approach to investigating perceptions and preferences around this kind of products that was established by Grasso and Jaworska (3) in the study of online reviews of hybrid meat products. In doing so, this study contributes to the growing body of research which utilises a combination of qualitative, projective techniques with quantitative methods to gain a more holistic understanding of consumers’ perceptions, preferences and attitudes (14), here specifically in relation to novel hybrid meat products before and after co-creation.

## 2. Data and methods

Grasso et al. (8) outline the process of participant recruitment for the questionnaire that was put to consumers in the United Kingdom, Spain, and Denmark, including an element in which consumers “co-created” their own hybrid meat product, in order to identify willingness to try (WTT) and willingness to buy (WTB) hybrid meat products in each of these countries. In this questionnaire, consumers were presented with a hybrid meat product and asked to provide four words that came to mind based on their first impression; they were asked to repeat this exercise when presented with the hybrid meat product that they had developed as part of the co-creation task. While studies focussing on general terms often ask participants to note down three words, most research on food and food behaviour that used the word association technique required more words mostly four [e.g., (11, 12)] to account for the diversity of dimensions and aspects that people associate with food and give participants a bit more “space” to report on those. We followed this parameter in this study too and selected four as the number of words to write down.

For the purpose of this study, “word” in the context of a consumer’s response refers to any single response from a user regardless of its length; thus, a “word” may be anything from the individual words *healthy*, *gross*, or *awareness* to phrasal responses such as *environmentally-friendly* or *a bit weird*. In some instances, consumers provided longer clausal responses such as *good way to get more vegetables* or *I wouldn’t buy it* and those were considered too.

Subsequently, all lexical items were categorised according to their dominant semantic meanings. Seven main categories were identified including: Evaluation, Sensory, Production, Emotion, Diets, Quality, Ethics, and Other. Given the wide range of words and phrases provided by consumers, each main category was then divided into relevant subcategories of meanings. The process of classifying the words and phrases into the categories was conducted iteratively and often by considering the context, that is, the other words that were provided in the response. Because of the explicit nature of the task, the meanings of most words and phrases were unambiguous and easy to categorise. In some ambiguous cases, the Oxford English Dictionary was consulted (16). An interrater was employed to classify 30% of the data with words and phrases from each category and subcategory. The agreement rate was generally high above 85%; any inconsistencies were resolved on the spot, and changes adopted.

A total of 802 participants in each of the United Kingdom and Demark, and 801 in Spain, provided words in English, Spanish, or Danish. Some respondents in Spain and Denmark provided their responses in English; for those who responded in Spanish and Danish, words were translated for the purposes of analysis. Theoretically, the total possible word yield for each language was 3,208 (3,204 for Spanish); however, certain words were rejected from the analysis for one of the following reasons:

• they appeared to be nonsense or gibberish, or were perhaps the result of a typo so severe that the original meaning could not be determined;
• the same word occurred more than once in a single set of four-word responses from a single consumer, i.e., the consumer repeated a word;
• a phrasal/clausal response was spread across more than one field—for example, one user responded with *I*, *don’t*, *like*, and *it*, which were amalgamated as the single response *I don’t like it*.

The total number of words for each language in each condition—both before (−CC) and after (+CC) the co-creation of a hybrid meat product—is provided in Table 2. Note that clausal responses were significantly (*p* < 0.001) more common in responses from Danish consumers when compared to English and Spanish, accounting for the majority of the variation in number of words for that language since therefore multiple fields were more often amalgamated into one response.

**TABLE 2:** Words provided by participants in each language before and after co-creation of a hybrid meat product.

	English *n* = 802	Spanish *n* = 801	Danish *n* = 802
−CC	3,151	3,168	3,022
+CC	3,153	3,177	3,026

Throughout the analysis that follows, statistical significance was determined through treatment of the figures above as six individual corpora and a calculation of the log-likelihood value. This standard measure of statistical significance in corpus linguistics takes the frequency of a particular phenomenon in one corpus or “body” of words and compares it to another, relative to the total size of each corpus; the log-likelihood is therefore a probability statistic that measures the likelihood of frequency differences between two or more corpora as occurring due to chance. This is then compared to a table of critical values to determine the statistical significance—or lack thereof—of the difference in frequency between the corpora. More information on the use of statistical analyses in corpus linguistics can be found in McEnery and Hardie (17).

## 3. Perceptions and evaluations of hybrid meat products

Following the process of classifying the words in accordance with their meanings, the following main semantic categories (with subcategories) emerged: Evaluation, Sensory, Production, Emotion, Diets, Quality, Ethics and Other. The full set of categories is provided in Table 3, with examples for each. As Table 3 shows, consumers referred to a range of dimensions when prompted to provide four words about hybrid-meat products before (−CC) and after (+CC) co-creation. While categories such as Sensory or general Evaluation are expected, there were also other aspects that were deemed relevant by consumers such as Ethics and varied dimensions of Quality and Emotion. This suggests that, when it comes to new hybrid food products, consumers do not just focus on one aspect—for example, only the sensory experience—but consider a variety of issues related to food production and consumption, including ethical conduct and sustainability, that are not often clearly communicated by food manufacturers.

**TABLE 3:** Frequency of consumer responses by category both before and after co-creation for each language.

Semantic category	English		Spanish		Danish	
	**−CC**	**+CC**	**−CC**	**+CC**	**−CC**	**+CC**
Evaluation	3,151	3,153	3,168	3,177	3,022	3,026
Sensory	644	806	425	707	454	626
Production	320	361	451	471	406	440
Emotion	305	308	333	280	444	387
Diets	174	27	211	41	113	18
Quality	1,006	1,030	1,217	1,148	900	881
Ethics	217	151	254	189	347	262
Other	219	161	131	109	144	153

Some interesting differences can be observed regarding the responses provided by the three national groups of consumers. Whereas, and as expected, Evaluation was relevant for all, words pointing to Emotion and Ethics were more often employed by Danish respondents, especially before the co-creation task, but this also remained quite relevant after the co-creation task. This suggests that Danish consumers might place more value on ethical and sustainable food production and consumption, and tend to express their preferences in a more emotional way. In addition, dimensions involved in Production of the hybrid meat products (including ingredients and nutritional value) seem to be more important for Spanish and Danish respondents than those from the United Kingdom.

In the sections that follow, each of the categories listed in Table 4 is taken in turn and noteworthy observations are made about some or all of the subcategories therein, noting statistical significance where appropriate to demonstrate a reasonable conclusion that the findings relate to real-world differences between consumers’ perceptions before co-creation of a hybrid meat product versus after. In the tables, the following notation has been used alongside +CC figures where appropriate to indicate the level of the statistically significant difference from the −CC figures: *p < 0.05; **p < 0.01; ***p < 0.001; ****p < 0.0001.

**TABLE 4:** Categories and subcategories with examples.

Semantic category	Semantic subcategory	Examples^a^
Evaluation	Positive	*great-tasting*, *I like it*, *recommend*
	Negative	*greasy*, *not enough*, *repellent*
	Neutral	*basil*, *recipe*, *umami*
	Ambiguous	*challenging*, *no meat*, *surprising*
Sensory	Appearance	*colourful*, *unappealing*
	Consistency	*chewy*, *stodgy*
	Smell	*fragrant*, *rank*
	Taste	*bland*, *yum*
	Texture	*crispy*, *rubbery*
Production	Ingredients	*falafel*, *olives*
	Nutrition	*high in vitamins*, *low salt*
	Process	*homemade*, *wok food*
	Side effects	*diuretic*, *flatulence*
Emotion	Confusion	*bewildered*, *I don’t know what to say*
	Expectation	*doubt*, *sceptical*
	Intention	*I wouldn’t buy it*, *yes please*
	Interest	*don’t care*, *intriguing*
	Mood	*heart-warming*, *shocking*
	Trust	*misleading*, *trick*
Diets	Allergies	*gluten*, *lactose-free*
	Disorders	*anorexia*, *diabetes*
	Religion	*halal*, *sin*
	Veganism	*herbivorous*, *vegan shit*
	Vegetarianism	*not as good as vegetarian*, *veggie burger*
	Weight loss	*fat-free*, *slimming*
Quality	Freshness	*fresh*, *perishable*
	Healthiness	*digestible*, *immune-building*
	Innovation	*inventive*, *novelty*
	Naturalness	*laboratory*, *pretend*
	Potential	*profitable*, *waste of time*
	Prestige	*niche*, *snobbery*
	Price	*cheaper*, *overpriced*
Ethics	Animal welfare	*animal rights*, *cruelty-free*
	Environment	*environmentally-friendly*, *polluting*
	General	*kinder*, *more ethical*
Other	Brands	*Heck*, *Jamie Oliver*
	Choice	*available*, *rarity*
	Convenience	*flexible*, *practical*
	Trendiness	*fashionable*, *politically-correct*

^a^Examples include some of the most frequent words as well as phrasal responses.

### 3.1. Evaluation

The category of evaluation is of particular note, not only in isolation but especially when cross-referenced with figures from the other categories and subcategories that follow. Since this category exemplifies the spirit of the words that consumers gave, it highlights whether they are viewing the product—and the specific features of the product identified by the subcategories—as positive or negative. Neutral words (those with no clear positive or negative association) were also identified, as well as words that were ambiguous in that they were likely to have a positive or negative association that was impossible to determine without further context; for example, the adjective *optimistic* may imply a positive outlook, or it may be a negative warning as to the potential for the product to be successful. The total number of words in each Evaluation category used by speakers in each language before and after co-creation are presented in Table 5.

**TABLE 5:** Frequency figures for subcategories of evaluation.

Semantic subcategory	English		Spanish		Danish	
	**−CC**	**+CC**	**−CC**	**+CC**	**−CC**	**+CC**
Positive	1,346	2,390****	1,376	2,448****	1,153	1,992****
Negative	1,038	308****	876	229****	993	368****
Neutral	594	366	751	410	725	537
Ambiguous	173	89	165	90	151	129
Totals	3,151	3,153	3,168	3,177	3,022	3,026

For all three languages, the number of positive words increased and the number of negative words decreased significantly (*p* < 0.001) following the co-creation task; the number of neutral and ambiguous words also decreased slightly in each case, indicating that there was a direct shift from negative to positive words after co-creation.

Table 6 shows the top 15 words used by the three groups of consumers before and after co-creation, with neutral and negative words highlighted in light and dark grey respectively. It is clear from this that the amount of negative words has been reduced, but it is also interesting to note the way in which specific words change in evaluation, sometimes from a negative word to its direct positive opposite. In most cases, before co-creation, consumers perceived the hybrid meat products as expensive, but this perception was diminished after the task, with Spanish consumers even using the word *cheap*, suggesting that they are now more amenable to paying more for the products (the possible interpretation of this word as meaning “poorly-made” seems unlikely given its contrast with *expensive* before co-creation). This seems plausible in light of the increased attention to nutrition, as indicated by the more frequent use of words such as *nutritious*, *nourishing*, and *balanced*, which made it to the top words following the co-creation task; the implication here is that consumers perceive a hybrid meat product as better value for money once they are more informed about its nutritional value. It is also noteworthy that lexical items pointing to positive taste experiences (*tasty*, *delicious*, *appetising*) remain in the top words in both conditions, whereas those that are negative (*tasteless*, *insipid*, *bland*, *boring*) disappear from this list.

**TABLE 6:** Top 15 words given by consumers in each of the three languages both before (−CC) and after (+CC) co-creation, with neutral and negative words highlighted.

	English				Spanish				Danish			
	**−CC**		**+CC**		**−CC**		**+CC**		**−CC**		**+CC**	
	**Word**	**Fq.**	**Word**	**Fq.**	**Word**	**Fq.**	**Word**	**Fq.**	**Word**	**Fq.**	**Word**	**Fq.**
**1**	Healthy	237	Tasty	450	Healthy	361	Tasty	509	Healthy	177	Healthy	318
**2**	Tasty	141	Healthy	363	Tasty	102	Healthy	508	New	77	Delicious	145
**3**	Tasteless	116	Different	132	Vegan	91	Nutritious	165	Tasteless	70	Tasty	136
**4**	Expensive	98	Nutritious	104	Expensive	82	Appetising	74	Expensive	68	Exciting	101
**5**	Different	92	New	67	Insipid	80	Good	69	Boring	67	New	90
**6**	Vegan	82	Interesting	65	Weird	77	Natural	59	Taste	67	Tasteful	69
**7**	Bland	80	Healthier	59	Vegetarian	63	Different	57	Healthier	54	Taste	65
**8**	Healthier	54	Good	41	Flavour	59	Original	48	Exciting	51	Healthier	55
**9**	Fake	52	Unique	41	Natural	59	Balanced	44	Environmentally-friendly	49	Different	54
**10**	Vegetarian	51	Ethical	39	Different	51	Cheap	41	Different	48	Environmentally-friendly	51
**11**	Boring	43	Nice	39	Ecological	51	Delicious	36	Environment	45	Interesting	44
**12**	Interesting	43	Spicy	36	Artificial	44	Flavour	34	Delicious	43	Nourishing	44
**13**	Sustainable	43	Delicious	34	Vegetable	42	Weird	34	Vegetarian	39	Expensive	41
**14**	New	40	Nutritional	30	Fake	41	Expensive	33	Vegan	37	OK	40
**15**	Good	38	Fun	29	Sustainable	39	Novel	33	Interesting	33	Easy	34

In isolation, this could be considered a mixed result for the hybrid meat product market: on the one hand, it implies that consumers see such products very positively once they are more familiar with them and know what is included in the product or have some choice of what “goes in.” On the other hand, this result highlights that informing or educating consumers about hybrid meat products and their ingredients is a key factor in determining consumer attitudes, since their first impressions before the co-creation were considerably more negative.

Evaluation was cross-referenced with the demographic of gender to determine any link to consumers’ attitudes. Men were found to respond with more negative words than women both before and after co-creation for all three languages, to a statistically significant degree of *p* < 0.05 or greater. Accordingly, female consumers gave more positive responses than male consumers before co-creation in all three languages (*p* < 0.01 or greater), and after co-creation for Spanish and Danish (*p* < 0.001); in English, the difference in the number of positive words between men and women was not found to be statistically significant.

Evaluation was also cross-referenced with further demographic information regarding the age range, education level, and purchasing responsibility of the respondents, but no significant differences were observed; for this reason and those of space, these demographics were not further considered in the analysis.

### 3.2. Sensory

Although this category looked at all senses relevant to the consumption of food, the only subcategory that achieved a number of responses of any note was taste (Table 7). For this category, once again there was a clear and significant (*p* < 0.001) increase in positive words (*delicious*, *tastes good*, *well-seasoned*) and decrease in negative words (*gross*, *bland*, *weird flavours*) for all three languages following the co-creation of a hybrid meat product. This is particularly interesting since the consumers have not of course had the opportunity to taste the products between the two times at which they provided their responses; this implies that consumers’ perceptions of taste can be “imagined” or primed by features of the product and its ingredients that they are exposed to during the task.

**TABLE 7:** Frequency figures for subcategories of sensory.

Semantic subcategory	English		Spanish		Danish	
	**−CC**	**+CC**	**−CC**	**+CC**	**−CC**	**+CC**
Appearance	26	15	29	23	45	32
Consistency	31	43	29	34	38	30
Smell	5	2	3	5	9	9
Taste	528	729	328	628	331	533
Texture	54	17	36	17	31	22
Totals	644	806	425	707	454	626

### 3.3. Production

The subcategory of nutrition (Table 8) was of particular note in this category, showing the same trend that positive attitudes to nutrition (*rich in vitamins*, *nutritious*, *without saturated fat*) increased significantly (*p* < 0.001) for all languages following co-creation. In this case, there were very few negative (*fatty*, *lack of vitamins*) or ambiguous (*fat content*, *without carbohydrates*) words given relating to nutrition in any language or condition, and the handful of neutral words (*nutrition*, *protein*) did not change in any significant way.

**TABLE 8:** Frequency figures for subcategories of production.

Semantic subcategory	English		Spanish		Danish	
	**−CC**	**+CC**	**−CC**	**+CC**	**−CC**	**+CC**
Ingredients	213	117****	326	189****	318	239****
Nutrition	88	236	102	266	63	185
Process	18	8	20	10	22	16
Side effects	1	0	3	6	3	0
Totals	320	361	451	471	406	440

There was a significant (*p* < 0.001) decrease in the amount that consumers in all languages discussed the ingredients of a hybrid meat product following its co-creation. While the overwhelming majority of words were neutral in nature, since they were merely references to the specific ingredients that could be found in the product (*pepper*, *chickpeas*), it shows that there is a shifting of focus among consumers of the topics that are of importance to them once they have engaged in a co-creation task. Indeed, cross-referencing the subcategories of ingredients and nutrition demonstrated that, prior to co-creation, responses relating to ingredients were significantly (*p* < 0.001) more prevalent than those relating to nutrition, but that this statistical significance is reversed following co-creation—that is, responses relating to nutrition were significantly (*p* < 0.001) more prevalent than those relating to ingredients. This suggests that, by becoming more intimately involved with the process of creating a hybrid meat product, consumers are prompted to think more carefully about its nutrition rather than the top-level ingredients that it contains; this may have important implications for the impact of co-creation on consumers’ understanding of nutrition and the healthiness of their diets.

### 3.4. Emotion

Consumer confusion (Table 9), as indicated by words that overtly expressed confusion (*bewildered*, *complicated*) or those that implied it through a lack of knowledge (*why*, *I don’t know*), formed only a small number of responses both before and after co-creation; nevertheless, there was a statistically significant decrease in its prevalence for English (*p* < 0.01), Spanish (*p* < 0.05), and Danish (*p* < 0.025). The number of responses in the subcategory of interest also decreased, although it may be that some of these, such as *intriguing*, indicated a desire to know more about hybrid meat products which was somewhat sated by the end of the co-creation task.

**TABLE 9:** Frequency figures for subcategories of production.

Semantic subcategory	English		Spanish		Danish	
	**−CC**	**+CC**	**−CC**	**+CC**	**−CC**	**+CC**
Confusion	55	26***	41	24*	50	28**
Expectation	8	3	14	12	26	17
Intention	70	98	99	158	81	96
Interest	111	94	40	26	143	82
Mood	22	66	51	34	75	136
Trust	39	21****	88	26****	69	28****
Totals	305	308	333	280	444	387

Consumers’ perceptions relating to intention were of particular note in this category: those that indicate positive intention (*appetising*, *I’d try*, *want to buy*)—that is, a desire to buy or eat the product—rose significantly (*p* < 0.001) across all three languages following co-creation, and, correspondingly, those that indicated negative intention (*avoid*, *no thanks*, *I’m not eating that*) fell significantly (*p* < 0.01), again across all languages. It is clear then that the co-creation task undertaken by consumers had a positive effect on their WTT and WTB that prompts further investigation.

A relatively small number of words relating negatively to trust (*deceptive*, *scam*, *swindle*) were found before co-creation, forming the majority of the words relating to this subcategory. Following the co-creation task, such words were found to have decreased in use significantly (*p* < 0.001) for all three languages, although there was little or no rise in the number of positive words relating to trust; rather, the topic seemed to no longer be of focus to consumers once they had undertaken the task and understood how hybrid meat products are made and the science behind them.

Only a small number of consumer responses were related to mood, and there was a general tendency toward those that were positive in meaning (*delightful*, *exciting*, *inspirational*). These positive mood words increased significantly (*p* < 0.001) for English and Danish consumers, but there was no change to be found for Spanish consumers.

### 3.5. Diets

Overall there were very few words for any of the diet-related subcategories (Table 10). In this category, consumers spoke most often about vegetarianism (*semi-vegetarian*, *veggie sausages*) and veganism (*vegan-mad veganish*), and there was a statistically significant (*p* < 0.001) decrease in words relating to these topics following co-creation for all three languages. Since the majority of words for vegetarianism and veganism were neutral in any case, it is likely that this topic simply became irrelevant for many consumers once they had learned more about hybrid meat products and the fact that they do contain meat (and are hence not vegetarian or vegan products). There is the wider implication here that the proper marketing of these products to highlight their meat content is likely to increase the number of consumers who are willing to try them as they will not be so easily dismissed by meat-eaters as suitable only for vegetarian or vegan diets.

**TABLE 10:** Frequency figures for subcategories of diets.

Semantic subcategory	English		Spanish		Danish	
	**−CC**	**+CC**	**−CC**	**+CC**	**−CC**	**+CC**
Allergies	3	0	1	0	6	0
Disorders	0	0	1	1	1	0
Religious	1	0	0	0	2	0
Vegan	86	4****	101	9****	53	2****
Vegetarian	73	18****	72	11****	49	10****
Weight loss	11	5	36	20	2	6
Totals	174	27	211	41	113	18

### 3.6. Quality

One important focus for many consumers—and indeed for manufacturers—was the price of the hybrid meat products (Table 11). For English consumers, the number of responses related to this topic decreases following co-creation, but for Spanish and Danish consumers it remains a topic of interest. However, by cross-referencing with evaluation scores, it is clear that the number of negative responses relating to price (*expensive*, *overpriced*, *unaffordable*) decreased significantly for English and Spanish (*p* < 0.001) as well as Danish (*p* < 0.025) consumers. An increase in positive words relating to price (*cheaper*, *economical*, *worth the price*) was significant only for Spanish consumers (*p* < 0.001).

**TABLE 11:** Frequency figures for subcategories of quality.

Semantic subcategory	English		Spanish		Danish	
	**−CC**	**+CC**	**−CC**	**+CC**	**−CC**	**+CC**
Freshness	23	22	10	8	8	16
Healthiness	341	466	466	609	292	431
Innovation	197	302****	220	227	207	195
Naturalness	205	75	309	114	197	70
Potential	80	76	69	57	79	69
Prestige	16	11	21	18	7	8
Price	144	78	122	115	110	92
Totals	1,006	1,030	1,217	1,148	900	881

The healthiness of the products was a topic of a great amount of focus for consumers, and for all three languages over 94% of words relating to healthiness were positive in nature (*good for you*, *wellbeing*, *it’s healthier*) even before the co-creation task. Despite this, positive responses concerning healthiness increased significantly (*p* < 0.001) to over 98% for all languages following the task. This is in contrast to some extent to the concept of naturalness, which was more often perceived negatively by many consumers both before and after the co-creation task. Nevertheless, the degree to which consumers responded with negative words about naturalness (*artificial*, *fake*, *Frankenstein*) decreased significantly (*p* < 0.001) for all languages following co-creation and the increased understanding of how hybrid meat products are developed.

The perception of innovation of the hybrid meat products was addressed by a number of consumers and was done so with an overwhelmingly positive outlook (*progressive*, *pioneering*, *futuristic*); this remained constant for Spanish and Danish following the co-creation task, while for English it increased further to a statistically significant extent (*p* < 0.001).

### 3.7. Ethics

For words relating to ethical considerations (Table 12)—mostly restricted to matters of the environment and animal welfare—consumers generally gave positive responses (*more climate-friendly*, *animal-friendly*, *no guilt*). Following co-creation, these generally seemed to decline, although the shift was not majorly significant and did not result in any increase in the number of negative words relating to ethical matters. In general, then, ethical topics were of less interest to consumers following co-creation, likely because of the increase in focus of matters relating specifically to the product that they had created—its naturalness, healthiness, affordability, etc.—and because, once an ethical issue had been registered upon the first viewing of the product, it did not seem necessary to repeat this fact after co-creation.

**TABLE 12:** Frequency figures for subcategories of ethics.

Semantic subcategory	English		Spanish		Danish	
	**−CC**	**+CC**	**−CC**	**+CC**	**−CC**	**+CC**
Animal welfare	20	6	13	6	38	28
Environment	152	72	218	141	282	206
General	45	73	23	42	27	28
Totals	217	151	254	189	347	262

### 3.8. Other

The subcategory of convenience (Table 13) shows that many consumers were concerned with how easy the products would be to prepare and cook, but in general this was the case only following co-creation. While topics such as ingredients, animal and environmental welfare, and vegetarianism/veganism were more frequent before the task, once they had created their own hybrid meat product many consumers focused more frequently on the level of convenience of the products, doing so in a positive way (*easy to cook*, *straightforward*, *helpful*), and increasingly so to a significant degree (*p* < 0.05) in all languages.

**TABLE 13:** Frequency figures for subcategories of other.

Semantic subcategory	English		Spanish		Danish	
	**−CC**	**+CC**	**−CC**	**+CC**	**−CC**	**+CC**
Brand	53	2	3	1	7	2
Choice	43	38	53	45	59	58
Convenience	30	82	17	45	21	70
Trendiness	93	39****	58	18****	57	23****
Totals	219	161	131	109	144	153

Some consumers also commented on the trendiness of the product; this subcategory produced a number of words that were ambiguous in nature, since the perception that something is “trendy” or “fashionable” is not always considered a positive attribute. Following the co-creation task, the number of words relating to trendiness fell significantly (*p* < 0.001) in all languages, and this was primarily from those that were evaluated as negative (*gimmicky*, *faddish*, *bandwagon*) or ambiguous (*modern*, *fashionable*, *politically-correct*). In the same way as figures for the subcategories of vegetarianism and veganism, it seems that perceptions of the products as being limited to a specific group or type of person are decreased once the co-creation task has been completed.

## 4. Conclusion

It is clear that the process of co-creation results in much more favourable perceptions of hybrid meat products. Following co-creation, consumers have been shown to have a more positive perception of the nutritional value of a product, as well as more trust and stronger intentions to try or buy them. Regarding taste—a factor of great importance to consumers—the majority of negative considerations of taste disappear following co-creation; while this could imply that consumers might decide they would like the product after all, it might also suggest that those consumers who would not personally find the product appealing can at least appreciate the positive aspects of the product as an available option in the supermarket. In addition, while the majority of consumers consider the products healthy even before co-creation, following the task these perceptions are significantly increased and any concerns about the unnaturalness of the hybrid meat products are diminished.

While it is possible that there is an effect here caused by the fact that the consumer has had a hand in the products development (a kind of “I made it” effect), the key difference following co-creation is that the consumer now has a better understanding of what the product is and how it is developed. This is exemplified by the words that the consumers used to describe them, particularly through (1) a decrease in words relating to confusion, (2) a greater focus on nutritional value over basic ingredients, and (3) a decrease in words such as *vegetarian* and *vegan* that highlight misunderstandings of the nature of a hybrid meat product. It is reasonable to conclude therefore that a major barrier to the positive perception of hybrid meat products is a lack of understanding about their nature and the processes involved in their development. This finding can be seen in a positive light: to rephrase a paragraph from section 3.1, although there is a considerable proportion of consumers who perceive hybrid meat products negatively, there is hope in that these perceptions can be significantly minimalized through education. However, the package may not be the place to undertake this education, since consumer opinions can be formed very quickly and on very little information (18); instead, it is likely that consumers will need to have a positive perception of a hybrid meat product before they enter the supermarket. Future work should focus on understanding the best way to communicate to consumers what hybrid meat products are and what their potential benefits might be. Another challenge lies in the fact that hybrid meat products currently are somewhere “in-between,” so while they surely do not belong to the vegan and vegetarian isles in the supermarkets, they should be given a dedicated and somehow highlighted section in the meat aisle to point out their differences from the meat-only products available.

Perhaps owing to the fact that hybrid meat products are an innovation to most markets, few differences were found between the three languages studied. Nevertheless, those differences that were found may be of importance: Danish consumers focused significantly more on ethical issues and gave more responses in relation to emotion, while they and Spanish consumers were both more concerned with the production process (including both the ingredients and the nutritional value) compared to English consumers. These differences in focus suggest that different marketing strategies should be employed in each market in order to successfully appeal to the consumers therein.

The results of this study show that co-creation matters for consumers’ perceptions of certain aspects of hybrid meat products, and therefore any new launches should be carefully co-created with consumers from the outset. The food industry should use co-creation tools more as they can provide valuable insights before products are developed and launched into the market, and therefore increase the chances of successful, competitive and tailored products on the shelves. In any case, the need for greater education regarding the nature of hybrid meat products is clear, and should be the first priority.

### 4.1. Limitations and further study

As a pioneering examination of the topic of attitudes to hybrid meat products in this way, this study has generally taken an approach that is broader than it is deep. It covers a large number of categories of words given by participants to analyse the overall trends in relation to positivity and negativity, and how these are influenced by an online co-creation task that helps educate consumers about the nature of hybrid meat products. A key area for future study, therefore, is to examine these trends in greater detail, with reference to demographic qualities of the respondents and any differences to be found therein. Although categories such as gender, age, education level and purchasing responsibility were considered initially, no significant differences were identified on the surface level and so these were not pursued further. It is highly anticipated that this type of study could be repeated with greater emphasis on demographic characteristics of the consumers now that the most significant concerns have been identified and that the positive benefits of the co-creation task on consumer attitudes has been established. Given that food choices often have to do with lifestyle preferences, further research would benefit from operationalising and including lifestyle as a factor alongside established demographic variables such as age and gender.

The socioeconomic situations in the United Kingdom, Spain, and Denmark have been addressed in this study, but, needless to say, the door has been opened for the same types of examination of further situations. These could be expanded within the same countries, such as a comparison of attitudes within each of the Home Nations of the United Kingdom, or could be widened to consider the same languages as spoken in alternative socioeconomic areas, such as the United States and Canada, Mexico and South America, and Greenland. Finally, there is great scope to broaden the study into further languages, in particular those spoken in Europe within the same socioeconomic bloc, such as French, German, and Swedish.

The approach taken here has combined qualitative with quantitative techniques, employing corpus-linguistic methods to analyse attitudes based on frequency counts that rise and fall following the co-creation task. Further studies could benefit from this approach, but it would also be invaluable to employ more qualitative research methodologies such as focus groups with consumers in each country and language, as these may yield more comprehensive and nuanced results. Beyond lexical prompts, such focus groups could involve sensory stimuli to tap into more immediate corporeal perceptions of hybrid meat products. Although the technique of spontaneous free word associations has been identified as a powerful tool to understand consumers’ perceptions, expectations and their food behaviour, future studies could complement word associations with other projective techniques, such as those involving, for example, story techniques and completion tasks to gain more holistic insights into consumers perceptions of hybrid meat products [e.g., (14)].

## Data availability statement

The original contributions presented in this study are included in the article/supplementary material, further inquiries can be directed to the corresponding author.

## Ethics statement

The studies involving human participants were reviewed and approved by University of Reading Ethics Committees. The patients/participants provided their written informed consent to participate in this study.

## Author contributions

SG collected the data for the study and contributed to refining the final version. CR organised the database and completed the statistical analysis. SJ and CR performed data categorisation and analysis and wrote the first draft. All authors contributed to the article and approved the submitted version.
